# Extraction of Information Related to Adverse Drug Events from Electronic Health Record Notes: Design of an End-to-End Model Based on Deep Learning

**DOI:** 10.2196/12159

**Published:** 2018-11-26

**Authors:** Fei Li, Weisong Liu, Hong Yu

**Affiliations:** 1 Department of Computer Science University of Massachusetts Lowell Lowell, MA United States; 2 Center for Healthcare Organization and Implementation Research Bedford Veterans Affairs Medical Center Bedford, MA United States; 3 Department of Medicine University of Massachusetts Medical School Worcester, MA United States; 4 School of Computer Science University of Massachusetts Amherst, MA United States

**Keywords:** adverse drug event, deep learning, multi-task learning, named entity recognition, natural language processing, relation extraction

## Abstract

**Background:**

Pharmacovigilance and drug-safety surveillance are crucial for monitoring adverse drug events (ADEs), but the main ADE-reporting systems such as Food and Drug Administration Adverse Event Reporting System face challenges such as underreporting. Therefore, as complementary surveillance, data on ADEs are extracted from electronic health record (EHR) notes via natural language processing (NLP). As NLP develops, many up-to-date machine-learning techniques are introduced in this field, such as deep learning and multi-task learning (MTL). However, only a few studies have focused on employing such techniques to extract ADEs.

**Objective:**

We aimed to design a deep learning model for extracting ADEs and related information such as medications and indications. Since extraction of ADE-related information includes two steps—named entity recognition and relation extraction—our second objective was to improve the deep learning model using multi-task learning between the two steps.

**Methods:**

We employed the dataset from the Medication, Indication and Adverse Drug Events (MADE) 1.0 challenge to train and test our models. This dataset consists of 1089 EHR notes of cancer patients and includes 9 entity types such as Medication, Indication, and ADE and 7 types of relations between these entities. To extract information from the dataset, we proposed a deep-learning model that uses a bidirectional long short-term memory (BiLSTM) conditional random field network to recognize entities and a BiLSTM-Attention network to extract relations. To further improve the deep-learning model, we employed three typical MTL methods, namely, hard parameter sharing, parameter regularization, and task relation learning, to build three MTL models, called HardMTL, RegMTL, and LearnMTL, respectively.

**Results:**

Since extraction of ADE-related information is a two-step task, the result of the second step (ie, relation extraction) was used to compare all models. We used microaveraged precision, recall, and F1 as evaluation metrics. Our deep learning model achieved state-of-the-art results (F1=65.9%), which is significantly higher than that (F1=61.7%) of the best system in the MADE1.0 challenge. HardMTL further improved the F1 by 0.8%, boosting the F1 to 66.7%, whereas RegMTL and LearnMTL failed to boost the performance.

**Conclusions:**

Deep learning models can significantly improve the performance of ADE-related information extraction. MTL may be effective for named entity recognition and relation extraction, but it depends on the methods, data, and other factors. Our results can facilitate research on ADE detection, NLP, and machine learning.

## Introduction

### Background

An adverse drug event (ADE) is an injury resulting from a medical drug intervention [1]. Previous studies reported that ADEs could account for up to 41% of all hospital admissions [2,3]. An ADE may cause a prolonged length of stay in the hospital and increase the economic burden [4]. The annual cost of ADEs for a 700-bed hospital is approximately $5.6 million [5]. Moreover, the total number of iatrogenic deaths can reach nearly 800,000 per year, which is higher than the death rate of heart disease or cancer [6]. In 2013, medical error, including ADEs, is the third most-common cause of death in the United States [7]. Therefore, ADE detection and report are crucial for pharmacovigilance and drug-safety surveillance [8,9].

Two methods are usually used to detect and report ADE. In premarketing surveillance, ADEs can be discovered during phase III clinical trials for drug development. In postmarketing surveillance, ADEs are discovered by patients and physicians using the Food and Drug Administration (FDA) Adverse Event Reporting System (FAERS). These traditional methods are limited by the number of participants [10], underreporting [11], and missing patterns of drug exposure [12]; for example, underreporting is a known issue in FAERS and may occur due to several reasons. First, the objective and content of the report in FAERS change over time, which may confuse physicians and the general public. Second, patients may choose not to mention some reactions, due to which practitioners fail to report them. Third, ADEs with long latency or producing unusual symptoms may be unrecognized. Other reasons may include payments from pharmaceutical companies to doctors [13] and inefficient communication between patients, physicians, and pharmacists. Recently, the FDA made the FAERS data available through a public dashboard [14]. Since anyone can view ADE reports online, this venture may help the FDA receive feedback to improve the FAERS.

Many researchers have used other resources to identify ADEs, such as biomedical publications [15,16], social media [17,18], and electronic health record (EHR) notes [19-21]. The ADEs extracted from these resources are an important complement to traditional ADE-surveillance systems. However, manual collection of ADEs from these data is laborious [22]. As such, the use of computer systems is a good choice to automatically detect ADEs, but may fail since these data are often unstructured text. Therefore, natural language processing (NLP) techniques are employed for this significant task [15,20,21,23].

From the viewpoint of NLP, ADE detection is covered under the task of information extraction, which includes ADE extraction as well as information related to ADE, such as medications and indications. Extraction of ADE-related information can be casted as a two-step pipeline. The first step is named entity recognition (NER) [24], which recognizes a string of text as an entity (eg, medication or ADE) that is predefined by dataset annotators. The second step is relation extraction (RE) [15], which is a model that determines whether two entities have a specific relation (eg, ﻿medication and ADE).

Previous studies employed traditional machine-learning techniques [15,16,23,24] such as condition random field (CRF) [25] or support vector machine (SVM) [26]. Recently, deep learning attracted much attention in NLP due its numerous advantages such as better performances and less feature engineering compared to other systems [27,28]. However, only a few studies have addressed extraction of ADE-related information via deep learning. Since ADE detection can be divided into two tasks (ie, NER and RE), it is logical to incorporate multi-task learning (MTL) [29] into ADE detection. However, only limited prior work has investigated the impact of MTL on ADE detection.

### Relevant Literature

#### Adverse Drug Event Detection

Since ADEs play an important role in drug-safety surveillance, ADE detection receives increasing attention from both the federal regulation and the research community. Besides the official reporting system FAERS, there are other databases that collect data on known ADEs, such as the Comparative Toxicogenomics Database [30] and SIDER [31]. Various resources have been used to detect ADEs, such as biomedical publications [15,16], social media [17,18], and electronic health record (EHR) notes [19-21]. For example, Gurulingappa et al [16] leveraged medical case reports to build a corpus in order to support drug-related adverse effects. Wei et al [15] organized a challenge task to extract chemical-induced disease relations from the literature and created an annotated corpus from 1500 articles. With respect to the methods, both supervised and unsupervised methods were used. Ramesh et al [32] developed a supervised machine-learning model to extract adverse event entities from FAERS narratives. Xu and Wang [33] used a semisupervised bootstrapped method to construct a knowledge base for the drug-side-effect association. Liu et al [34] proposed a causality-analysis model based on structure learning for identifying factors that contribute to adverse drug reactions. Yildirim et al [35] applied the k-mean algorithm to identify adverse reactions. Xu et al [23] used SVM to extract ADEs between sentence-level and document-level drug-disease pairs. Recently, Munkhdalai et al [21] attempted to use deep learning to address ADE extraction, but their model was not end-to-end and relied on the entities.

**Figure 1:**
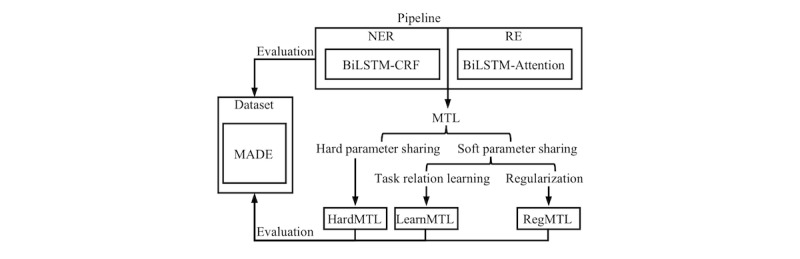
Study overview. NER: named entity recognition. RE: relation extraction. BiLSTM: bidirectional long short-term-memory. CRF: conditional random field. MTL: multi-task learning. MADE: Medication, Indication, and Adverse Drug Events. HardMTL: multi-task learning model for hard parameter sharing. RegMTL: multi-task learning model for soft parameter sharing based on regularization. LearnMTL: multi-task learning model for soft parameter sharing based on task-relation learning.

#### Named Entity Recognition

NER is a standard task that has been studied for many years in NLP [25]. Many researchers made important contributions to dataset construction including the GENIA corpus [36], the NCBI disease corpus [37], and the ShARe/CLEF eHealth evaluation [38]. Early studies addressed NER by diverse approaches such as rule-based [39] and machine-learning approaches [40-42], among which CRF-based approaches predominated. For example, Campos et al [40] presented a CRF model to recognize biomedical names, which achieved state-of-the-art performance at the time by incorporating rich features. Tang et al [43] modified the label scheme of CRF to make it be capable of recognizing disjoint clinical concepts. However, such approaches need many efforts for feature engineering. Recently, a bidirectional LSTM (BiLSTM) model [44,45] was proposed and became a popular method for NER. In the biomedical domain, Jagannatha and Yu [20] used such a model to detect medical events from EHR notes.

#### Relation Extraction

RE has been widely studied, and some typical RE corpora in the biomedical domain include the 2010 i2b2/VA challenge [46] and BioCreative V CDR task [15]. Early work used rules and NLP structures such as dependency trees [47] and coreference chains [48] to help extract relations. Others usually leveraged machine-learning approaches such as SVM [49,50] and structured learning [51]. As deep learning developed, researchers proposed a number of neural network models to handle RE [52,53]. Our study is related to the joint or end-to-end entity and RE, which allows performance of NER and RE simultaneously. Miwa and Bansal [54] proposed an end-to-end model based on the sequence and tree LSTM. Similarly, Mehryary et al [55] proposed an end-to-end system to extract information about bacteria and their habitats.

#### Multi-Task Learning

MTL [29] refers to training the model for multiple related tasks. It is widely used in artificial intelligence research such as computer vision [56] and NLP [57]. Learning these tasks simultaneously may improve the performance as compared to learning the tasks individually. Prior MTL studies mostly focused on homogeneous MTL that consists of tasks with only one type such as classification or regression [58]. Some of their tasks are closely related, such as cross-lingual [59] and synthetic data [60]. Based on a previous study [58], MTL can be roughly divided into two categories—hard and soft parameter sharing. For hard parameter sharing, the lower layers are shared among multiple tasks and each task has its own higher layer [54]. For soft parameter sharing, each task has its own model with its own parameters. There are some representative methods for soft parameter sharing such as regularization [59] or learning task relations [56].

### Objective

Since only a few prior studies have addressed end-to-end detection of ADE via deep learning, we aimed to design a two-step pipeline model that consists of two submodels: a BiLSTM [61] CRF [25] network for NER and a BiLSTM-Attention [62] network for RE. Since extraction of ADE-related information includes two steps, it is possible to study the impact of MTL on NER and RE. However, only limited prior work has focused on MTL with such heterogeneous and loosely related tasks. Therefore, our second objective was to fill this gap by proposing three MTL models and comparing them with the pipeline model. An overview of this study is shown in Figure 1.

## Methods

### Deep Learning Pipeline Model

#### BiLSTM-CRF Submodel for NER

**Figure 2:**
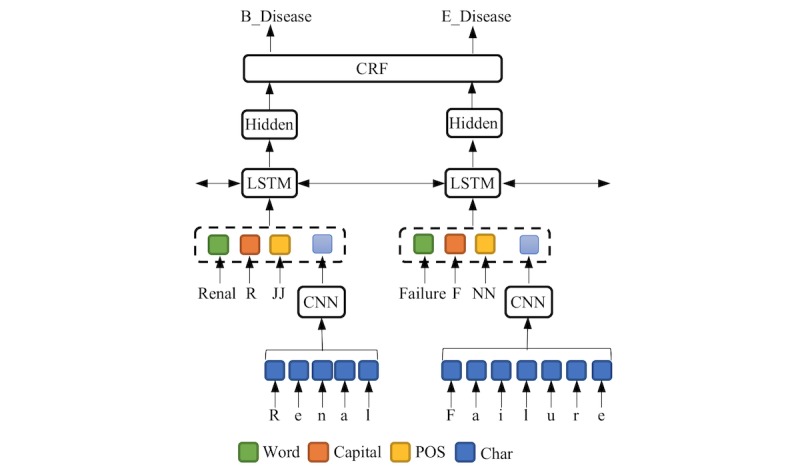
NER submodel. For simplicity, here we use “Renal Failure” to illustrate the architecture. For “Renal,” the word feature is “Renal,” the capital feature of the initial character is “R,” the POS feature is “JJ,” and the character representation is generated from CNN. NER: named entity recognition. CNN: convolutional neural network. CRF: condition random field. LSTM: long short-term memory. CNN: convolutional neural network. POS: part of speech.

Our NER submodel is presented in Figure 2. We extended the state-of-the-art BiLSTM-CRF model [44,45] by enriching its features. For a sentence, we extracted four kinds of features for each token, namely, its word, whether the initial character is capital, its part-of-speech (POS) tag, and its character representation. We employed a convolutional neural network to obtain character representation. After the token representations are obtained by concatenating the features, we fed them into a bidirectional LSTM layer to learn the hidden representations. Subsequently, the hidden representations were input into the CRF layer to determine the optimal labels for all the tokens in the sentence. For labels, we use the BMES (Begin, Middle, End, Singular) label scheme [45] plus entity types. For example, the label of the token “Renal” is “B_Disease.” The details of the BiLSTM-CRF submodel for NER are provided in Multimedia Appendix 1.

#### BiLSTM-Attention Submodel for RE

**Figure 3:**
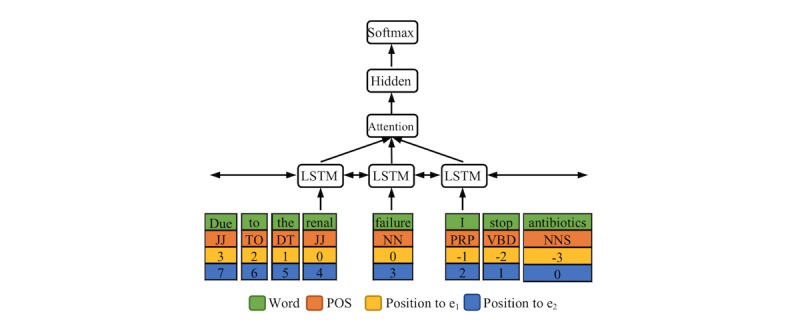
RE submodel. The target entities are “renal failure” (e_1_) and “antibiotics” (e_2_). Positions represent token distances to the target entities. RE: relation extraction. LSTM: long short-term memory. POS: part of speech.

Our RE submodel is presented in Figure 3. A relation instance can be considered as a token sequence and two target entities. Here, the token sequence did not necessarily have to be one sentence, as we could also extract intersentence relations. For each token, we extracted four kinds of features, namely, its word, its POS tag, and the position embeddings [63]. Here, the character representation was not used, because it reduced the performance in our preliminary experiments. Similar to the case for NER, we employed a BiLSTM layer to generate the hidden representations. Subsequently, the attention method [62] was used to obtain context features.

Because only context features may not be enough to capture the semantic relation, we also employed other features for concision, which are not shown in Figure 3. Considering previous work [21], these features included words of two target entities, types of two target entities, the token number between two target entities, and the entity number between two target entities. Like the word or POS embeddings, these features can be represented as vectors. Therefore, the output layer considers the concatenation of all these features to determine the relation of target entities. The details of the BiLSTM-Attention submodel for RE are provided in Multimedia Appendix 2.

### Multi-Task Learning Models

In this section, we propose three MTL models: one model used hard parameter sharing [54] and two models used soft parameter sharing, namely, regularization [59] and task relation learning [56].

#### HardMTL

**Figure 4:**
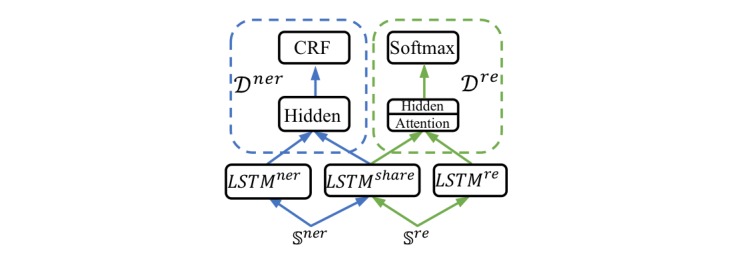
The high-level view of HardMTL. For conciseness, “LSTM” indicates a BiLSTM layer, and the layers above the BiLSTM layer are denoted as *D^new^* and *D^re^*. The forward procedures for an NER instance and an RE instance are indicated by blue and green arrow lines, respectively. HardMTL: multi-task learning model for hard parameter sharing. LSTM: long short-term-memory. BiLSTM: bidirectional long short-term-memory. CRF: conditional random field. NER: named entity recognition. RE: relation extraction.

Our MTL model for hard parameter sharing is presented in Figure 4. We employed the shared-private architecture [64] to make each submodel of each task retain its private parts and share some parts for multi-task learning. The NER and RE submodels had their own BiLSTM layers, namely, *LSTM*^*ner*^ and *LSTM*^*re*^, and shared a BiLSTM layer, *LSTM*^*share*^. During training, the shared BiLSTM layer *LSTM*^*share*^ was used by both the NER and RE submodels, so that it was tuned during the back-propagation by both submodels. Therefore, the model was able to learn useful knowledge from both tasks. The details of the HardMTL model are provided in Multimedia Appendix 3.

#### RegMTL

**Figure 5:**
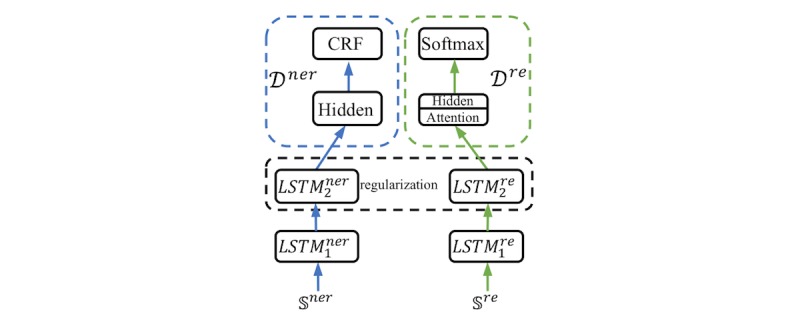
The high-level view of RegMTL. *LSTM_1_^ner^* and *LSTM_2_^ner^* indicate the first and second BiLSTM layers of the NER model. *LSTM_1_^re^* and *LSTM_2_^re^* indicate the first and second BiLSTM layers of the RE model. NER: named entity recognition. RE: relation extraction. RegMTL: multi-task learning model for soft parameter sharing based on regularization. BiLSTM: bidirectional long short-term-memory. CRF: conditional random field. LSTM: long short-term-memory.

Our first MTL model for soft parameter sharing was based on regularization, and its architecture is presented in Figure 5. With reference to previous studies [59,60], we employed the L2 regularization in order to encourage the parameters of the NER and RE submodels to be similar instead of sharing some parts in the networks. Two BiLSTM layers were considered because different inputs of the NER and RE submodels lead to different dimensions of the first BiLSTM layer; therefore, L2 regularization of the parameters of the first BiLSTM layer was computationally intractable. We resolved this issue by performing L2 regularization in the second BiLSTM layer. The details of the RegMTL model are provided in Multimedia Appendix 3.

#### LearnMTL

**Figure 6:**
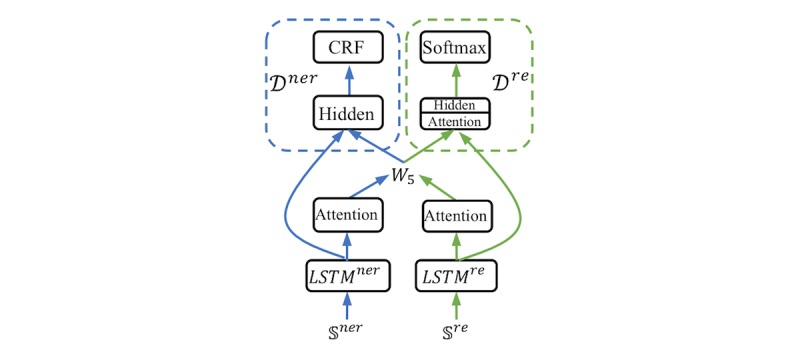
The high-level view of LearnMTL. LearnMTL: multi-task learning model for soft parameter sharing based on task-relation learning. CRF: conditional random field. LSTM: long short-term-memory.

Our second MTL model for soft parameter sharing was based on task relation learning [56], and its architecture is illustrated in Figure 6. After generating hidden representations from the BiLSTM and attention layers, we used a linear layer, *W*_*5*_, to exchange information between the NER and RE submodels. To utilize task-specific and shared information, the concatenation of hidden representations of the BiLSTM and information exchange layers was fed into the upper decoders *D*^*ner*^ and *D*^*re*^. The details of the LearnMTL model are provided in Multimedia Appendix 3.

### Dataset

We used the MADE dataset from the MADE1.0 challenge for detecting medications and ADEs from EHR notes [65]. It consists of 1089 EHR notes of patients with cancer, from which data for 18 common Protected Health Information aspects were removed according to ﻿the Health Insurance Portability and Accountability Act. The dataset was separated into 876 notes for training and 213 notes for testing. In this dataset, the annotators annotated not only ADEs, but also other ADE-related information. They predefined 9 entity types, namely, Medication, Indication, Frequency, Severity, Dosage, Duration, Route, ADE, and SSLIF (any sign, symptom, and disease that is not an ADE or Indication). In addition, they predefined 7 relation types between these entity types, namely, ﻿Dosage-Medication, Route-Medication, Frequency-Medication, Duration-Medication, Medication-Indication, Medication-ADE, and Severity-ADE.

## Results

The experimental settings used to obtain these results are provided in Multimedia Appendix 4.

### Comparison Between Our Best Model and Existing Systems

We compared our models with the top three systems in the MADE1.0 challenge. Chapman et al [66] used CRF for NER and random forest for RE. Specifically, two random forest models were used—one for detecting whether relations exist between entities and the other for classifying what specific relation type exists. Xu et al [67] used BiLSTM-CRF for NER with word, prefix, suffix, and character features. For RE, they used SVM and designed features such as positions, distances, bag of words, and bag of entities. Dandala et al [68] also used BiLSTM-CRF for NER, but they input different features into the model such as words, POS tags, and characters. For RE, they employed the BiLSTM-Attention model that takes tokens, entity types, and positions as input.

Full neural systems ([68] and our study) achieve better performances with the MADE dataset than with other systems (Table 1). Although the main methods between the study of Dandala et al [68] and our study are similar, our model is much better, as it significantly improved the F1 for RE by 5%. The reasons for this superiority may be that we used more features than previous work, such as capital information and entity words, and our model attained approximately 0.8% improvement in F1 from MTL.

### Comparison Between the Pipeline and MTL Models

The HardMTL model outperforms other models, achieving an F1 of 84.5% in NER and 66.7% in RE (Table 2); the pipeline model ranks second, with F1 values of 84.1% and 65.9%, respectively. The RegMTL model obtains the best recall in both NER (84.5%) and RE (63.6%). Surprisingly, the most-complex MTL model LearnMTL ranked last.

In our experiments, HardMTL successfully boosted the NER F1 by 0.4% (*P*=.003) and the RE F1 by 0.8% (*P*=.01), but RegMTL and LearnMTL failed to boost the performances. Thus, the effectiveness of different MTL methods depends on the selected tasks and data. For heterogenous and loosely related tasks such as NER and RE, it is more difficult for MTL to be effective.

### Performance of Each Entity Type

Table 3 shows the performance of each entity type. Medication and Route (both F1>90%) were easier to recognize than other types. In contrast, ADE is the most-difficult type to recognize (F1=55%). Other entity types with lower performances included Indication and Duration.

### Performance of Each Relation Type

Table 4 shows the performance of each relation type. Medication-ADE relations are the most-difficult type to extract (F1=45.5%). Severity-ADE ranks second (F1=54.1%), followed by Duration-Medication (F1=59.5%). In contrast, Route-Medication and Dosage-Medication extraction are relatively easier, with F1>80%.

### Comparison Between the Pipeline Model and MedEx System

MedEx [69] is an end-to-end system used to identify medications and their attributes such as routes and dosages. Therefore, the final results of MedEx correspond to our results for extracting 4 kinds of relations: Route-Medication, Dosage-Medication, Duration-Medication, Frequency-Medication. Table 5 compares MedEx with our model. Our model significantly outperformed MedEx, which demonstrates that our model is a competitive system in this domain.

**Table 1:** Comparison of our model with the existing systems in the Medication, Indication, and Adverse Drug Events dataset. The microaveraged F1s of relation extraction are shown according to the official evaluation report.

System	Named entity recognition	Relation extraction	F1
Chapman et al [66]	CRF^a^	Random forest	59.2
Xu et al [67]	BiLSTM^b^-CRF	Support vector machine	59.9
Dandala et al [68]	BiLSTM-CRF	BiLSTM-Attention	61.7
Our Best (HardMTL^c^)	BiLSTM-CRF	BiLSTM-Attention	66.7

^a^CRF: conditional random field

^b^BiLSTM: bidirectional long short-term memory

^c^HardMTL: multi-task learning model for hard parameter sharing

**Table 2:** Performances (%) of the pipeline and multi-task learning models. The values presented are the means of 5 runs of each model. The microaveraged P, R, and F1s of all entity or relation types are shown.

Method	Entity recognition			Relation extraction		
	P	R	F1	P	R	F1
Pipeline	85.0	83.2	84.1	69.8	62.4	65.9
HardMTL^a^	85.0	84.1	84.5	70.2	63.6	66.7
RegMTL^b^	84.5	84.5	84.5	66.7	63.6	65.1
LearnMTL^c^	84.5	82.8	83.6	67.2	61.5	64.2

^a^HardMTL: multi-task learning model for hard parameter sharing

^b^RegMTL: multi-task learning model for soft parameter sharing based on regularization

^c^LearnMTL: multi-task learning model for soft parameter sharing based on task relation learning

**Table 3:** Performance (%) of each entity type.

Entity type	P	R	F1
Medication	91.1	92.0	91.3
Indication	65.4	64.8	64.8
Frequency	87.1	86.5	86.3
Severity	84.6	84.7	84.7
Dosage	87.9	86.4	88.0
Duration	75.3	76.6	77.6
Route	91.6	91.9	91.9
Adverse drug events	59.5	57.6	55.4
SSLIF^a^	83.9	84.8	84.9

^a^SSLIF: any sign, symptom, and disease that is not an ADE or Indication

**Table 4:** Performance (%) of each relation type.

Relation type	P	R	F1
Severity-Adverse drug events	55.0	54.4	54.1
Route-Medication	81.0	82.5	82.1
Medication-Indication	53.9	52.5	52.9
Dosage-Medication	80.9	79.8	81.0
Duration-Medication	60.3	63.7	59.5
Frequency-Medication	77.7	78.6	78.4
Medication-Adverse drug events	50.4	47.6	45.5

**Table 5:** Results (%) of comparisons between our pipeline model and the MedEx system.

Entity type	MedEx system			Pipeline model		
	P	R	F1	P	R	F1
Route-Medication	71.9	47.9	57.5	81.0	82.5	82.1
Dosage-Medication	29.7	3.5	6.2	80.9	79.8	81.0
Duration-Medication	25.5	15.6	19.4	60.3	63.7	59.5
Frequency-Medication	52.5	36.2	42.8	77.7	78.6	78.4

## Discussion

### Principal Findings

Existing systems usually selected a two-step pipeline to address ADE-related information extraction: recognizing entities and extracting relations. BiLSTM-CRF is the most-popular model for NER, whereas the selections of RE models are mixed. All our models outperformed the existing systems in the MADE1.0 challenge, which may be because of the following reasons: First, our models benefited from deep learning that is able to learn better from the data. Second, we enriched the features of deep learning models; therefore, our model outperformed the system [68] that used similar deep learning models as ours.

For MTL, we found that the model using hard parameter sharing (HardMTL) performed better than the other two models using soft parameter sharing (RegMTL and LearnMTL) and that the most complex MTL model, LearnMTL, performed the worst in our data. Our results are not surprising, as different MTL methods depend on different tasks and data [54,56,59]. Overall, MTL more difficult between heterogeneous and loosely related tasks such as NER and RE.

In our experiments, the entity type “ADE” and relation type “Medication-ADE” were the most difficult information to be extracted. Based on our analysis, this is not only due to a lack of training data, but also the intrinsic character of ADEs. ADEs are often implicit in the context without any obvious pattern, which negatively affects the model (Example 1 in Multimedia Appendix 5). In contrast, some entity or relation types with obvious patterns (eg, Medication-Dosage) are easier to identify (Example 2 in Multimedia Appendix 5).

Finally, we found that the performance improved when we used the pretrained word embeddings in the biomedical domain [70] rather than those in the general domain. Furthermore, if the pretrained word embeddings were not tuned, our models would perform better. One likely reason for this is that such a method can alleviate the overfitting problem.

### Error Analysis

We randomly sampled hundreds of error instances of NER and RE. Through the manual analyses, we found several sources of errors. For NER, the major false-negative errors are due to long expressions of entities (Examples 3 and 4 in Multimedia Appendix 5). These entities (eg, IgG kappa monoclonal protein) include multiple words; therefore, it is difficult to detect their boundaries. Moreover, the major false-positive errors for NER occur because some entity types are incorrectly recognized as SSLIF (Examples 5 and 6 in Multimedia Appendix 5). This may be because the training instances of SSLIF are ≥10 times those of other entity types such as ADE. Thus, imbalanced data distribution may lead to certain bias of our models.

With respect to RE, the major false-negative errors are due to long distances between target entities (Example 9 in Multimedia Appendix 5). The relation of two entities can be expressed through ≥6 sentences in EHRs; therefore, our model may miss such relations in a long context. In addition, the major false-positive errors for RE occur because relation expressions exist in the instance, but are not related to the target entities (Examples 7 and 8 in Multimedia Appendix 5). For instance, in Example 7 of Multimedia Appendix 5, “His current therapy includes [thalidomide]_Entity1_ 50 mg a day for 2 weeks out of the month. He had been on Velcade, which was stopped secondary to increasing [peripheral neuropathy]_*Entity2*_*,* ” “peripheral neuropathy,” and “thalidomide” have no Medication-ADE relation, but the model incorrectly predicts their relation due to the words “secondary to.”

### Contributions

﻿

The main contributions of this work are as follows: (1) We proposed an up-to-date deep learning model to perform ADE-related information extraction in an end-to-end manner. Our model achieved new state-of-the-art performance, improving the F1 by 4.2% (absolute value). (2) To our knowledge, this is the first attempt to investigate the impact of MTL on two heterogeneous and loosely related tasks (ie, NER and RE). One of our MTL models further improved the F1 by 0.8% (absolute value). (3) ﻿Our manually annotated dataset—Medication, Indication, and Adverse Drug Events (MADE) [65]—will be publicly available to support the research on extraction of ADE-related information.

### Conclusions

We proposed a deep learning model to detect ADEs and related information. We also investigated MTL on two ADE-related tasks, NER and RE. Our models achieved state-of-the-art performance in an ADE-detection dataset. MTL can improve performance, but it depends on the methods and data used. In the future, we plan to evaluate our models with more related datasets.

## Appendix

Multimedia Appendix 1 BiLSTM-CRF submodel for NER. BiLSTM: bidirectional long short-term-memory. CRF: conditional random field. NER: named entity recognition.

Multimedia Appendix 2 BiLSTM-Attention submodel for RE. BiLSTM: bidirectional long short-term-memory. RE: relation extraction.

Multimedia Appendix 3 MTL models. MTL: multi-task learning.

Multimedia Appendix 4 Experimental settings.

Multimedia Appendix 5 Examples.

## Abbreviations

ADE: adverse drug event
BiLSTM: bidirectional long short-term-memory
CRF: conditional random field
EHR: electronic health record
FAERS: Food and Drug Administration Adverse Event Reporting System
LSTM: long short-term-memory
MADE: Medication, Indication, and Adverse Drug Events
MTL: multi-task learning
NER: named entity recognition
NLP: natural language processing
RE: relation extraction
POS: part of speech
SVM: support vector machine

## References

[1] Bates DW, Cullen DJ, Laird N, Petersen LA, Small SD, Servi D, Laffel G, Sweitzer BJ, Shea BF, Hallisey R (1995). Incidence of adverse drug events and potential adverse drug events. Implications for prevention. ADE Prevention Study Group. JAMA.

[2] Nebeker JR, Hoffman JM, Weir CR, Bennett CL, Hurdle JF (2005). High rates of adverse drug events in a highly computerized hospital. Arch Intern Med.

[3] Fattinger K, Roos M, Vergères P, Holenstein C, Kind B, Masche U, Stocker DN, Braunschweig S, Kullak-Ublick GA, Galeazzi RL, Follath F, Gasser T, Meier PJ (2000). Epidemiology of drug exposure and adverse drug reactions in two swiss departments of internal medicine. Br J Clin Pharmacol.

[4] Classen DC, Pestotnik SL, Evans RS, Lloyd JF, Burke JP (1997). Adverse drug events in hospitalized patients. Excess length of stay, extra costs, and attributable mortality. JAMA.

[5] Bates DW, Spell N, Cullen DJ, Burdick E, Laird N, Petersen LA, Small SD, Sweitzer BJ, Leape LL (1997). The costs of adverse drug events in hospitalized patients. Adverse Drug Events Prevention Study Group. JAMA.

[6] Null G, Dean C, Feldman M, Rasio D (2005). Death by Medicine. Journal of Orthomolecular Medicine.

[7] Makary MA, Daniel M (2016). Medical error-the third leading cause of death in the US. BMJ.

[8] Handler SM, Altman RL, Perera S, Hanlon JT, Studenski SA, Bost JE, Saul MI, Fridsma DB (2007). A systematic review of the performance characteristics of clinical event monitor signals used to detect adverse drug events in the hospital setting. J Am Med Inform Assoc.

[9] Kaushal R, Jha AK, Franz C, Glaser J, Shetty KD, Jaggi T, Middleton B, Kuperman GJ, Khorasani R, Tanasijevic M, Bates DW, BrighamWomen's Hospital CPOE Working Group (2006). Return on investment for a computerized physician order entry system. J Am Med Inform Assoc.

[10] Haas JS, Iyer A, Orav EJ, Schiff GD, Bates DW (2010). Participation in an ambulatory e-pharmacovigilance system. Pharmacoepidemiol Drug Saf.

[11] Edlavitch SA (1988). Adverse Drug Event Reporting. Arch Intern Med.

[12] Begaud B, Moride Y, Tubert-Bitter P, Chaslerie A, Haramburu F (2012). False-positives in spontaneous reporting: should we worry about them?. British Journal of Clinical Pharmacology.

[13] ProPublica.

[14] Kumar A (2018). The Newly Available FAERS Public Dashboard: Implications for Health Care Professionals. Hospital Pharmacy.

[15] Wei C, Peng Y, Leaman R, Davis AP, Mattingly CJ, Li J, Wiegers TC, Lu Z (2016). Assessing the state of the art in biomedical relation extraction: overview of the BioCreative V chemical-disease relation (CDR) task. Database (Oxford).

[16] Gurulingappa H, Rajput AM, Roberts A, Fluck J, Hofmann-Apitius M, Toldo L (2012). Development of a benchmark corpus to support the automatic extraction of drug-related adverse effects from medical case reports. J Biomed Inform.

[17] Lardon J, Abdellaoui R, Bellet F, Asfari H, Souvignet J, Texier N, Jaulent M, Beyens M, Burgun A, Bousquet C (2015). Adverse Drug Reaction Identification and Extraction in Social Media: A Scoping Review. J Med Internet Res.

[18] Abdellaoui R, Schück S, Texier N, Burgun A (2017). Filtering Entities to Optimize Identification of Adverse Drug Reaction From Social Media: How Can the Number of Words Between Entities in the Messages Help?. JMIR Public Health Surveill.

[19] Gurwitz JH (2003). Incidence and Preventability of Adverse Drug Events Among Older Persons in the Ambulatory Setting. JAMA.

[20] Jagannatha AN, Yu H (2016). Bidirectional RNN for Medical Event Detection in Electronic Health Records. Proc Conf.

[21] Munkhdalai T, Liu F, Yu H (2018). Clinical Relation Extraction Toward Drug Safety Surveillance Using Electronic Health Record Narratives: Classical Learning Versus Deep Learning. JMIR Public Health Surveill.

[22] Hurdle JF, Weir CR, Roth B, Hoffman J, Nebeker JR (2003). Critical gaps in the world's largest electronic medical record: Ad Hoc nursing narratives and invisible adverse drug events. AMIA Annu Symp Proc.

[23] Xu J, Wu Y, Zhang Y, Wang J, Lee H, Xu H (2016). CD-REST: a system for extracting chemical-induced disease relation in literature. Database (Oxford).

[24] Finkel J, Dingare S, Manning CD, Nissim M, Alex B, Grover C (2005). Exploring the boundaries: gene and protein identification in biomedical text. BMC Bioinformatics.

[25] Lafferty J, McCallum A, Pereira F (2001). Conditional Random Fields: Probabilistic Models for Segmenting and Labeling Sequence Data. https://repository.upenn.edu/cgi/viewcontent.cgi?article=1162&context=cis_papers.

[26] Vapnik VN (2009). The Nature of Statistical Learning Theory.

[27] LeCun Y, Bengio Y, Hinton G (2015). Deep learning. Nature.

[28] Rajkomar A, Oren E, Chen K, Dai AM, Hajaj N, Hardt M, Liu PJ, Liu X, Marcus J, Sun M, Sundberg P, Yee H, Zhang K, Zhang Y, Flores G, Duggan GE, Irvine J, Le Q, Litsch K, Mossin A, Tansuwan J, Wang D, Wexler J, Wilson J, Ludwig D, Volchenboum SL, Chou K, Pearson M, Madabushi S, Shah NH, Butte AJ, Howell MD, Cui C, Corrado GS, Dean J (2018). Scalable and accurate deep learning with electronic health records. Nature.

[29] Caruana R (1997). Multitask Learning. Machine Learning.

[30] Davis AP, Grondin CJ, Lennon-Hopkins K, Saraceni-Richards C, Sciaky D, King BL, Wiegers TC, Mattingly CJ (2015). The Comparative Toxicogenomics Database's 10th year anniversary: update 2015. Nucleic Acids Res.

[31] Kuhn M, Campillos M, Letunic I, Jensen LJ, Bork P (2010). A side effect resource to capture phenotypic effects of drugs. Mol Syst Biol.

[32] Polepalli RB, Belknap SM, Li Z, Frid N, West DP, Yu H (2014). Automatically Recognizing Medication and Adverse Event Information From Food and Drug Administration's Adverse Event Reporting System Narratives. JMIR Med Inform.

[33] Xu R, Wang Q (2014). Automatic construction of a large-scale and accurate drug-side-effect association knowledge base from biomedical literature. J Biomed Inform.

[34] Liu M, Cai R, Hu Y, Matheny ME, Sun J, Hu J, Xu H (2014). Determining molecular predictors of adverse drug reactions with causality analysis based on structure learning. J Am Med Inform Assoc.

[35] Yildirim P, Majnarić L, Ekmekci O, Holzinger A (2014). Knowledge discovery of drug data on the example of adverse reaction prediction. BMC Bioinformatics.

[36] Kim J, Ohta T, Tateisi Y, Tsujii J (2003). GENIA corpus--semantically annotated corpus for bio-textmining. Bioinformatics.

[37] Doğan RI, Leaman R, Lu Z (2014). NCBI disease corpus: a resource for disease name recognition and concept normalization. J Biomed Inform.

[38] Suominen H, Salanterä S, Velupillai S, Chapman W, Savova G, Elhadad N, Pradhan S, South B, Mowery D, Jones G, Leveling J, Kelly L, Goeuriot L, Martinez D, Zuccon G (2013). Overview of the ShARe/CLEF eHealth Evaluation Lab.

[39] Hanisch D, Fundel K, Mevissen H, Zimmer R, Fluck J (2005). ProMiner: rule-based protein and gene entity recognition. BMC Bioinformatics.

[40] Campos D, Matos S, Oliveira JL (2013). Gimli: open source and high-performance biomedical name recognition. BMC Bioinformatics.

[41] Hsu C, Chang Y, Kuo C, Lin Y, Huang H, Chung I (2008). Integrating high dimensional bi-directional parsing models for gene mention tagging. Bioinformatics.

[42] Zhou G, Zhang J, Su J, Shen D, Tan C (2004). Recognizing names in biomedical texts: a machine learning approach. Bioinformatics.

[43] Tang B, Chen Q, Wang X, Wu Y, Zhang Y, Jiang M, Wang J, Xu H (2015). Recognizing Disjoint Clinical Concepts in Clinical Text Using Machine Learning-based Methods. AMIA Annu Symp Proc.

[44] Lample G, Ballesteros M, Subramanian S, Kawakami K, Dyer C (2016). Neural Architectures for Named Entity Recognition.

[45] Yang J, Liang S, Zhang Y (2018). Design Challenges and Misconceptions in Neural Sequence Labeling. http://aclweb.org/anthology/C18-1327.

[46] Uzuner Ö, South BR, Shen S, DuVall SL (2011). 2010 i2b2/VA challenge on concepts, assertions, and relations in clinical text. J Am Med Inform Assoc.

[47] Fundel K, Küffner R, Zimmer R (2007). RelEx--relation extraction using dependency parse trees. Bioinformatics.

[48] Kilicoglu H, Rosemblat G, Fiszman M, Rindflesch TC (2016). Sortal anaphora resolution to enhance relation extraction from biomedical literature. BMC Bioinformatics.

[49] Lavergne T, Grouin C, Zweigenbaum P (2015). The contribution of co-reference resolution to supervised relation detection between bacteria and biotopes entities. BMC Bioinformatics.

[50] Airola A, Pyysalo S, Björne J, Pahikkala T, Ginter F, Salakoski T (2008). All-paths graph kernel for protein-protein interaction extraction with evaluation of cross-corpus learning. BMC Bioinformatics.

[51] Kordjamshidi P, Roth D, Moens M (2015). Structured learning for spatial information extraction from biomedical text: bacteria biotopes. BMC Bioinformatics.

[52] Luo Y, Cheng Y, Uzuner Ö, Szolovits P, Starren J (2018). Segment convolutional neural networks (Seg-CNNs) for classifying relations in clinical notes. J Am Med Inform Assoc.

[53] Verga P, Strubell E, McCallum A (2018). Simultaneously Self-Attending to All Mentions for Full-Abstract Biological Relation Extraction. http://aclweb.org/anthology/N18-1080.

[54] Miwa M, Bansal M (2016). End-to-end Relation Extraction using LSTMs on Sequences and Tree Structures.

[55] Mehryary F, Hakala K, Kaewphan S, Björne J, Salakoski T, Ginter F (2017). End-to-End System for Bacteria Habitat Extraction. http://www.aclweb.org/anthology/W17-2310.

[56] Misra I, Shrivastava A, Gupta A, Hebert M (2016). Cross-Stitch Networks for Multi-task Learning. http://openaccess.thecvf.com/content_cvpr_2016/papers/Misra_Cross-Stitch_Networks_for_CVPR_2016_paper.pdf.

[57] Collobert R, Weston J, Bottou L, Karlen M, Kavukcuoglu K, Kuksa P (2011). Natural language processing (almost) from scratch. The Journal of Machine Learning Research.

[58] Ruder S (2017). arXiv.

[59] Duong L, Cohn T, Bird S, Cook P (2015). Low Resource Dependency Parsing: Cross-lingual Parameter Sharing in a Neural Network Parser. http://anthology.aclweb.org/P/P15/P15-2139.pdf.

[60] Argyriou A, Evgeniou T, Pontil M (2006). Multi-task Feature Learning. https://papers.nips.cc/paper/3143-multi-task-feature-learning.pdf.

[61] Hochreiter S, Schmidhuber J (1997). Long Short-Term Memory. Neural Computation.

[62] Luong T, Pham H, Manning C (2015). Effective Approaches to Attention-based Neural Machine Translation. http://aclweb.org/anthology/D15-1166.

[63] Zeng D, Liu K, Lai S, Zhou G, Zhao J (2014). Relation Classification via Convolutional Deep Neural Network. http://www.aclweb.org/anthology/C14-1220.

[64] Chen X, Cardie C (2018). Multinomial Adversarial Networks for Multi-Domain Text Classification. http://www.aclweb.org/anthology/N18-1111.

[65] Jagannatha A, Liu F, Liu W, Yu H (2018). Overview of the First Natural Language Processing Challenge for Extracting Medication, Indication and Adverse Drug Events from Electronic Health Record Notes (MADE1.0). Drug Safety.

[66] Chapman A, Peterson K, Alba P, DuVall S, Patterson O (2018). Hybrid system for adverse drug event detection. http://proceedings.mlr.press/v90/chapman18a/chapman18a.pdf.

[67] Xu D, Yadav V, Bethard S (2018). UArizona at the MADE1.0 NLP Challenge. http://proceedings.mlr.press/v90/xu18a/xu18a.pdf.

[68] Dandala B, Joopudi V, Devarakonda M (2018). IBM Research System at MADE 2018: Detecting Adverse Drug Events from Electronic Health Records. http://proceedings.mlr.press/v90/dandala18a/dandala18a.pdf.

[69] Xu H, Stenner SP, Doan S, Johnson KB, Waitman LR, Denny JC (2010). MedEx: a medication information extraction system for clinical narratives. J Am Med Inform Assoc.

[70] Pyysalo S, Ginter F, Moen H, Salakoski T, Ananiadou S (2013). Distributional semantics resources for biomedical text processing. http://bio.nlplab.org/pdf/pyysalo13literature.pdf.

