# Supplementary material for: Extraction of Information Related to Adverse Drug Events from Electronic Health Record Notes: Design of an End-to-End Model Based on Deep Learning
Source: JMIR Med Inform. 2018 Nov 26;6(4):e12159. doi: 10.2196/12159 (PMC6288593; doi:10.2196/12159)
Supplement: Multimedia Appendix 1 [file medinform_v6i4e12159_app1.pdf]

Multimedia Appendix 1: BiLSTM-CRF Submodel for NER

Figure 1. NER submodel. For simplicity, here we use "Renal failure" to illustrate the architecture. For "Renal", its word feature is "Renal", its capital feature of the initial character is "R", its POS feature is "JJ" and the character representation is generated from CNN.

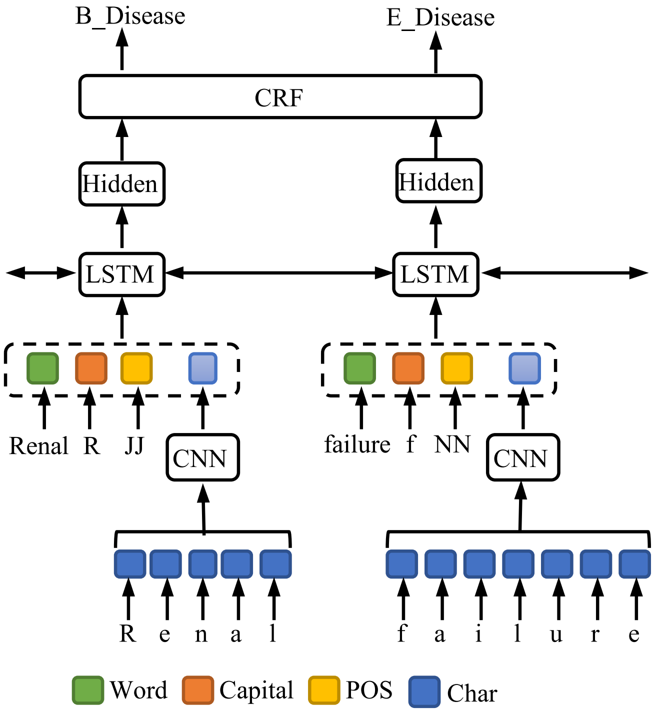

Our NER submodel is shown in Figure 1. We extended the state-of-the-art BiLSTM-CRF model [1,2] by enriching the features. Given a sentence $x^{ner} = \{x_1^{ner}, x_2^{ner}, \ldots, x_N^{ner}\}$, we extracted four kinds of features for each token x_n^{ner}, namely its word - w_n, whether the initial character is capital - cap_n, its part-of-speech (POS) tag - pos_n and its character representation - $char_n$. Since it is common to embed a feature into a vector in deep learning [1–3], we treat a feature and its corresponding embedding as interchangeable concepts for conciseness. Therefore, a token can be represented as:

$$x_n^{ner} = [w_n, cap_n, pos_n, char_n], \quad (1)$$

where [] denotes the concatenation operation. Note that we employed a convolutional neural network (CNN) to obtain the character representation $char_n$. Given a token x_n^{ner} that consists of M characters $\{c_1, c_2, \ldots, c_M\}$, the m-th convolution can be denoted as:

$$char'_m = W_1 \cdot [c_{m-1}, c_m, c_{m+1}], \quad (2)$$

where W_1 is the parameter matrix and "·" denotes the matrix multiplication. Throughout this paper, the bias vector is ignored for conciseness. For the head and rear characters, we

use padding characters to meet the convolutional size. Then the max-pooling is applied on all convolutional outputs to get the character representation $char_n$ of the token x_n^{ner}:

$$char_n \ = pooling(char_1', char_2', \ldots, char_M'). \qquad (3)$$

After the token representations x_n^{ner} is obtained, a LSTM unit takes it as input and outputs a vector h_n^{ner}. If bi-directional LSTM units are used, h_n^{ner} can be denoted as $[\overrightarrow{h_n^{ner}}, \overleftarrow{h_n^{ner}}]$. Then a score vector $z_n^{ner} \in \mathbb{R}^{L^{ner}}$ is computed as:

$$z_n^{ner} = W_2 \cdot h_n^{ner}, \qquad (4)$$

where W_2 is the parameter matrix and L^{ner} is the label size. Here we use the BMES (Begin, Middle, End, Singular) label scheme [2] plus entity types. For example, the label of the token "Renal" is "B_Disease".

Now the input sentence x^{ner} corresponds to a vector sequence $\{z_1^{ner}, z_2^{ner}, \ldots, z_N^{ner}\}$ which can also be considered as a matrix Z. For a label sequence $y^{ner} = \{y_1^{ner}, y_2^{ner}, \ldots, y_N^{ner}\}$, its score can be defined as:

$$s(x^{ner}, y^{ner}) = \sum_{n=1}^{N} Z_{n,y_n^{ner}} + \sum_{n=1}^{N} T_{y_{n-1}^{ner}, y_n^{ner}}, \qquad (5)$$

where $Z_{n,y_n^{ner}}$ denotes the score of the label y_n^{ner} of the n-th token, and $T_{y_{n-1}^{ner}, y_n^{ner}}$ indicates the score of a transition from the label y_{n-1}^{ner} to the label y_n^{ner}. During decoding, the label sequence with the highest score is selected as the prediction result. During training, the loss function is to minimize the negative log-likelihood of each instance in the training set $\mathbb{S}^{ner} = \{(x_i^{ner}, y_i^{ner})\}_I$:

$$\mathcal{L}(s, y_i^{ner}; \theta^{ner}) = -\log\left(\frac{\exp\left(s(x_i^{ner}, y_i^{ner})\right)}{\sum \exp\left(s(x_i^{ner}, \tilde{y}_i^{ner})\right)}\right), \qquad (6)$$

where (x_i^{ner}, y_i^{ner}) denotes the token and label sequences of the i-th instance and θ^{ner} denotes the parameters.

References

1. Lample G, Ballesteros M, Subramanian S, Kawakami K, Dyer C. Neural Architectures for Named Entity Recognition. Proc 2016 Conf North Am Chapter Assoc Comput Linguist [Internet] San Diego, California: Association for Computational Linguistics; 2016. p. 260–270. [doi: 10.18653/v1/N16-1030]

2. Yang J, Liang S, Zhang Y. Design Challenges and Misconceptions in Neural Sequence Labeling. Proc 27th Int Conf Comput Linguist Association for Computational Linguistics; 2018. p. 3879–3889.

3. Miwa M, Bansal M. End-to-end Relation Extraction using LSTMs on Sequences and Tree Structures. Proc 54th Annu Meet Assoc Comput Linguist [Internet] Association for Computational Linguistics; 2016. p. 1105–1116. [doi: 10.18653/v1/P16-1105]
