# Supplementary material for: Extraction of Information Related to Adverse Drug Events from Electronic Health Record Notes: Design of an End-to-End Model Based on Deep Learning
Source: JMIR Med Inform. 2018 Nov 26;6(4):e12159. doi: 10.2196/12159 (PMC6288593; doi:10.2196/12159)
Supplement: Multimedia Appendix 2 [file medinform_v6i4e12159_app2.pdf]

Multimedia Appendix 2: BiLSTM-Attention Submodel for RE

Figure 1. RE submodel. The target entities are "renal failure" (e_1) and "antibiotics" (e_2). Positions represent token distances to the target entities.

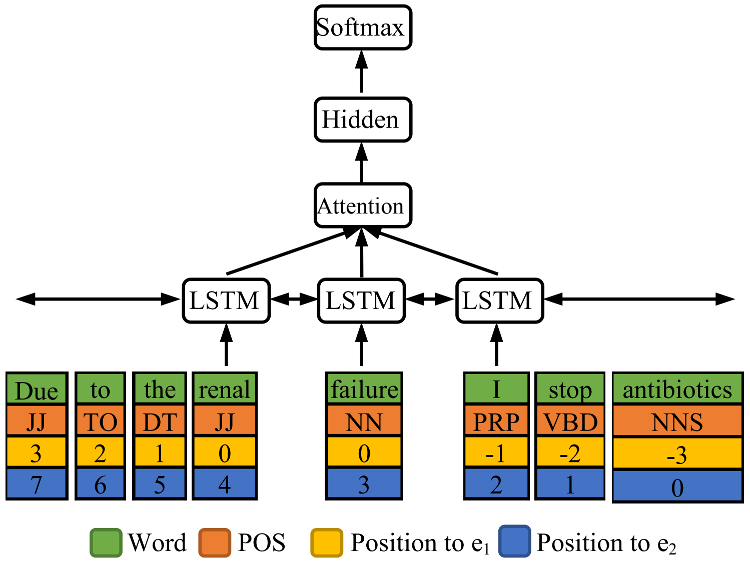

Our relation extraction submodel is shown in Figure 1. A relation instance can be considered as a token sequence $x^{re} = \{x_1^{re}, x_2^{re}, \ldots, x_N^{re}\}$ and two target entities e_1 and e_2. x^{re} is not necessary to be one sentence since we can also extract inter-sentence relations. x_n^{re} is a concatenation of four parts as shown below:

$$x_n^{re} = [w_n, pos_n, p_n^{e1}, p_n^{e2}], \qquad (1)$$

where p_n^{e1} and p_n^{e2} denote the position embeddings [1]. Here the character representation is not used since it hurts the performance in our preliminary experiments.

Similar to NER, we also employ bi-directional LSTM units to encode x_n^{re} of each token into a vector $h_n^{re} = [\overrightarrow{h_n^{re}}, \overleftarrow{h_n^{re}}]$. Then the attention method [2] is used to obtain the hidden vector h^{att} as below:

$$h^{att} = \sum_{n=1}^{N} \alpha_n \cdot h_n^{re}, \qquad (2)$$

where α_n is the weight of h_n^{re} , which is computed as $\alpha_n = softmax(W_3^T \cdot h_n^{re})$. W_3^T is the transposition of the attention vector W_3.

Because only h^{att} may be not enough to capture the semantic relation, we also employ other features that are not shown in Figure 1 for conciseness. Motivated by previous work [3], these features include: words of two target entities - ew_1 and ew_2; types of two target entities - et_1 and et_2; the token number between two target entities - tn; the entity number

between two target entities - *en*. Note that the inputs of the RE submodel are either annotated entities (during training) or predicted entities (during inference), so all the aforementioned features such as entity types or entity numbers are available at this time. Like the word or POS embeddings, these features can also be represented as vectors. Therefore, the output layer actually takes the concatenation of all these features as input:

$$z^{re} = W_4 \cdot \left[h^{att}, ew_1, ew_2, et_1, et_2, tn, en\right], \qquad (3)$$

where W_4 is the parameter matrix and $z^{re} \in \mathbb{R}^{L^{re}}$ is a score vector. L^{re} indicates the label size for relation extraction. During decoding, the relation label y^{re} with the highest score is selected as the prediction result. During training, the loss function is to minimize the negative log-likelihood of each instance in the training set $\mathbb{S}^{re} = \{(x_j^{re}, y_j^{re})\}_J$:

$$\mathcal{L}\left(s, y_j^{re}; \theta^{re}\right) = -\log\left(\frac{\exp\left(s\left(x_j^{re}, y_j^{re}\right)\right)}{\sum \exp\left(s\left(x_j^{re}, \tilde{y}_j^{re}\right)\right)}\right), \qquad (4)$$

where (x_i^{re}, y_i^{re}) denotes the token sequence and label of the j-th instance, and θ^{re} denotes the parameters. Here $s\left(x_j^{re}, y_j^{re}\right)$ can also be denoted as $(z^{re})_{y_j^{re}}$, i.e., the element of z^{re} that corresponds to y_j^{re}.

References

1. Zeng D, Liu K, Lai S, Zhou G, Zhao J. Relation Classification via Convolutional Deep Neural Network. Proc COLING 2014 Association for Computational Linguistics; 2014. p. 2335–2344.

2. Luong T, Pham H, Manning CD. Effective Approaches to Attention-based Neural Machine Translation. Proc 2015 Conf Empir Methods Nat Lang Process Association for Computational Linguistics; 2015. p. 1412–1421.

3. Munkhdalai T, Liu F, Yu H. Clinical Relation Extraction Toward Drug Safety Surveillance Using Electronic Health Record Narratives: Classical Learning Versus Deep Learning. JMIR Public Health Surveill 2018 Apr 25;4(2):e29. PMID:29695376
