# Supplementary material for: Extraction of Information Related to Adverse Drug Events from Electronic Health Record Notes: Design of an End-to-End Model Based on Deep Learning
Source: JMIR Med Inform. 2018 Nov 26;6(4):e12159. doi: 10.2196/12159 (PMC6288593; doi:10.2196/12159)
Supplement: Multimedia Appendix 3 [file medinform_v6i4e12159_app3.pdf]

Multimedia Appendix 3: MTL Models

HardMTL

Figure 1. The high-level view of HardMTL. For conciseness, "LSTM" indicates a BiLSTM layer, and the layers above the BiLSTM layer are denoted as $\mathcal{D}^{ner}$ and $\mathcal{D}^{re}$.

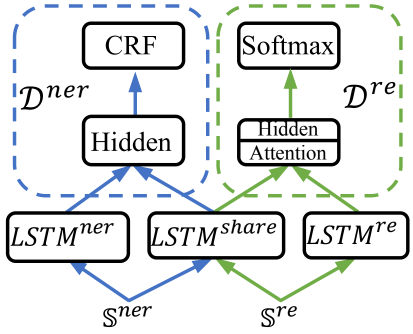

Our MTL model for hard parameter sharing is shown in Figure 1. We employ the shared-private architecture [1] to make each model of each task keep its private parts meanwhile share some parts for multi-task learning. Concretely, the NER and RE model have their own BiLSTM layers namely $LSTM^{ner}$ and $LSTM^{re}$, and they also share a BiLSTM layer $LSTM^{share}$. Since the layers above the BiLSTM layers determine how to decode hidden representations into prediction results, we call them $\mathcal{D}^{ner}$ and $\mathcal{D}^{re}$ for short.

The forward procedure for an NER instance is indicated by blue arrow lines. From bottom to top, an instance $(x_i^{ner}, y_i^{ner}) \in \mathbb{S}^{ner}$ is input into $LSTM^{ner}$ and $LSTM^{share}$, and we get two outputs, h^{ner} and h^{share}. Then the concatenation of h^{ner} and h^{share} is fed into $\mathcal{D}^{ner}$ and the loss is generated based on the objective function. After that, the parameters θ^{ner} of the NER submodel are updated based on the back-propagation algorithm from top to bottom. For each instance $(x_j^{re}, y_j^{re}) \in \mathbb{S}^{re}$, the training procedure is similar, and it is indicated by green arrow lines. Note that the shared BiLSTM layer $LSTM^{share}$ is used by both the NER and RE submodels, so it will be tuned during the back-propagation by both submodels. Therefore, it is able to learn useful knowledge from both tasks. The pseudo code for HardMTL training is shown in Algorithm 1.

Algorithm 1: HardMTL Training

Require: NER training set $\mathbb{S}^{ner} = \{(x_i^{ner}, y_i^{ner})\}_I$, RE training set $\mathbb{S}^{re} = \{(x_j^{re}, y_j^{re})\}_J$

1: repeat
2: for $(x_i^{ner}, y_i^{ner}) \in \mathbb{S}^{ner}$ do
3: $h^{ner} = LSTM^{ner}(x_i^{ner})$
4: $h^{share} = LSTM^{share}(w_i)$
5: $l^{ner} = \mathcal{L}\big(\mathcal{D}^{ner}([h^{ner}, h^{share}]), y_i^{ner}; \theta^{ner}\big)$
6: update θ^{ner} using ∇l^{ner}
7: for $(x_j^{re}, y_j^{re}) \in \mathbb{S}^{re}$ do
8: $h^{re} = LSTM^{re}(x_j^{re})$
9: $h^{share} = LSTM^{share}(w_j)$
10: $l^{re} = \mathcal{L}\big(\mathcal{D}^{re}([h^{re}, h^{share}]), y_j^{re}; \theta^{re}\big)$
11: update θ^{re} using ∇l^{re}
12: until convergence

h^{ner}, h^{share} and h^{re} denote vector sequences, e.g., $h^{ner} = \{h_1^{ner}, h_2^{ner}, \dots, h_N^{ner}\}$. w_i or w_j denotes the words in a training instance.

For each instance $(x_i^{ner}, y_i^{ner}) \in \mathbb{S}^{ner}$, $LSTM^{ner}$ takes x_i^{ner} as input and outputs h^{ner} (line 3). Note that the input of the NER and RE model is different, so the shared BiLSTM layer $LSTM^{share}$ should use words w_i as input (line 4) to meet the dimension need. Then the concatenation of h^{ner} and h^{share} is fed into $\mathcal{D}^{ner}$ and the loss l^{ner} is generated based on the objective function $\mathcal{L}$ (line 5). Finally, the parameters θ^{ner} are updated using the gradient ∇l^{ner} (line 6). For each instance $(x_j^{re}, y_j^{re}) \in \mathbb{S}^{re}$, the training procedure is similar (lines 7-11). Therefore, the shared BiLSTM layer $LSTM^{share}$ is tuned during training for both the NER and RE models, so it learns knowledge from both tasks.

RegMTL

Figure 2. The high-level view of RegMTL. $LSTM_1^{ner}$ and $LSTM_2^{ner}$ indicate the first and second BiLSTM layers of the NER model. $LSTM_1^{re}$ and $LSTM_2^{re}$ indicate the first and second BiLSTM layers of the RE model.

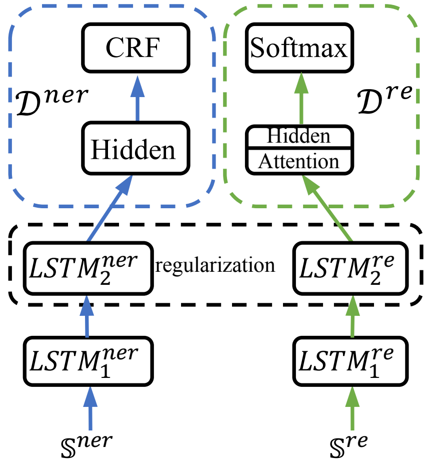

Our first MTL model for soft parameter sharing is based on regularization and its architecture is shown in Figure 2. Motivated by [2,3], we employ the L2 regularization to encourage the parameters of the NER and RE models to be similar instead of sharing some parts in the networks. The reason of two BiLSTM layers is that the different input of the NER and RE model leads to different dimensions of the first BiLSTM layer, while it is more convenient for the L2 regularization if the dimensions are identical. Therefore, we solve this problem by performing the L2 regularization in the second BiLSTM layer.

For each instance $(x_i^{ner}, y_i^{ner}) \in \mathbb{S}^{ner}$, the first BiLSTM layer $LSTM_1^{ner}$ takes x_i^{ner} as input and outputs h_1^{ner}. The second BiLSTM layer $LSTM_2^{ner}$ takes h_1^{ner} as input and outputs h_2^{ner}. Similar with HardMTL, the loss l^{ner} is generated on the objective function. Then the L2 regularization is applied on the parameters of the second BiLSTM layers and added into the loss, given by: $l^{ner} += \frac{1}{2}||\theta^{LSTM_2^{ner}} - \theta^{LSTM_2^{re}}||_2^2$. After that, the loss is back-propagated to update the parameters. For each instance $(x_j^{re}, y_j^{re}) \in \mathbb{S}^{re}$, the training procedure is similar. The pseudo code for RegMTL training is shown in Algorithm 2.

Algorithm 2: RegMTL Training

Require: NER training set $\mathbb{S}^{ner} = \{(x_i^{ner}, y_i^{ner})\}_I$, RE training set $\mathbb{S}^{re} = \{(x_j^{re}, y_j^{re})\}_J$

1: repeat
2: for $(x_i^{ner}, y_i^{ner}) \in \mathbb{S}^{ner}$ do
3: $h_1^{ner} = LSTM_1^{ner}(x_i^{ner})$
4: $h_2^{ner} = LSTM_2^{ner}(h_1^{ner})$
5: $l^{ner} = \mathcal{L}(\mathcal{D}^{ner}(h_2^{ner}), y_i^{ner}; \theta^{ner})$
6 $l^{ner} += \frac{1}{2} || \theta^{LSTM_2^{ner}} - \theta^{LSTM_2^{re}} ||_2^2$
7: update θ^{ner} using ∇l^{ner}
8: for $(x_j^{re}, y_j^{re}) \in \mathbb{S}^{re}$ do
9: $h^{re} = LSTM^{re}(x_j^{re})$
10: $h^{share} = LSTM^{share}(w_j)$
11: $l^{re} = \mathcal{L}\left(\mathcal{D}^{re}([h^{re}, h^{share}]), y_j^{re}; \theta^{re}\right)$
12: $l^{re} += \frac{1}{2} || \theta^{LSTM_2^{re}} - \theta^{LSTM_2^{ner}} ||_2^2$
13: update θ^{re} using ∇l^{re}
14: until convergence

$|| \cdot ||_2$ denotes the L2 regularization. $\theta^{LSTM_2^{ner}}$ and $\theta^{LSTM_2^{re}}$ denote the parameters of the second BiLSTM layers in the NER and RE models respectively.

For each instance $(x_i^{ner}, y_i^{ner}) \in \mathbb{S}^{ner}$, the first BiLSTM layer $LSTM_1^{ner}$ takes x_i^{ner} as input and outputs h_1^{ner} (line 3). The second BiLSTM layer $LSTM_2^{ner}$ takes h_1^{ner} as input and outputs h_2^{ner} (line 4). The loss l^{ner} is generated based on the prediction $\mathcal{D}^{ner}(h_2^{ner})$, the gold answer y_i^{ner} and the objective function $\mathcal{L}$. Then the L2 regularization is applied on the parameters of the second BiLSTM layers in the NER and RE models, i.e., $\theta^{LSTM_2^{ner}}$ and $\theta^{LSTM_2^{re}}$ (line 6). For each instance $(x_j^{re}, y_j^{re}) \in \mathbb{S}^{re}$, the training procedure is similar (lines 9-12).

LearnMTL

Figure 3. The high-level view of LearnMTL.

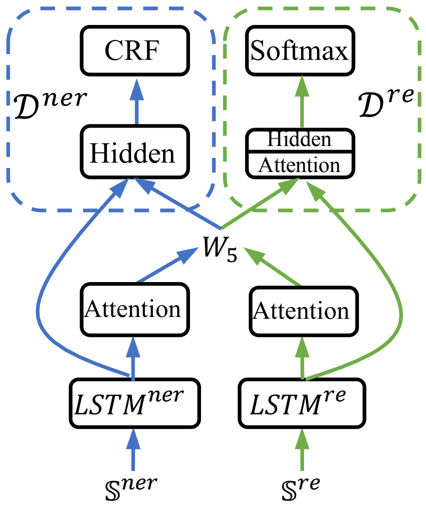

Our second MTL model for soft parameter sharing is based on task relation learning [4] and its architecture is shown in Figure 3. For a NER instance (x_i^{ner}, y_i^{ner}), the BiLSTM layer $LSTM^{ner}$ takes x_i^{ner} as input and outputs h^{ner}. Then we use the attention method to generate a vector $\tilde{h}^{ner}$. Similarly, h^{re} and $\tilde{h}^{re}$ can be generated from a RE instance $\left(x_j^{re}, y_j^{re}\right)$. $\tilde{h}^{ner}$ and $\tilde{h}^{re}$ can be considered as the information summarizations for the current NER and RE training instances respectively. Then the information is linearly combined via $\tilde{h} = W_5 \cdot [\tilde{h}^{ner}, \tilde{h}^{re}]$, where W_5 is the parameter matrix to learn task relations. To utilize task-specific and shared information, the concatenation $[h^{ner}, \tilde{h}]$ or $[h^{re}, \tilde{h}]$ is fed into the decoder $\mathcal{D}^{ner}$ or $\mathcal{D}^{re}$. Then the loss generation and parameter update are similar to aforementioned algorithms. The pseudo code for LearnMTL training is listed in Algorithm 3.

Algorithm 3: LearnMTL Training

Require: NER training set $\mathbb{S}^{ner} = \{(x_i^{ner}, y_i^{ner})\}_I$, RE training set $\mathbb{S}^{re} = \{(x_j^{re}, y_j^{re})\}_J$

1: repeat
2: for $(x_i^{ner}, y_i^{ner}) \in \mathbb{S}^{ner}, (x_j^{re}, y_j^{re}) \in \mathbb{S}^{re}$ do
3: $h^{ner} = LSTM^{ner}(x_i^{ner})$
4: $\tilde{h}^{ner} = attention(h^{ner})$
5: $h^{re} = LSTM^{re}(x_j^{re})$
6: $\tilde{h}^{re} = attention(h^{re})$
7: $\tilde{h} = W_5 \cdot [\tilde{h}^{ner}, \tilde{h}^{re}]$
8: $l^{ner} = \mathcal{L}\big(\mathcal{D}^{ner}([h^{ner}, \tilde{h}]), y_i^{ner}; \theta^{ner}\big)$
9: update θ^{ner} using ∇l^{ner}
10: $l^{re} = \mathcal{L}\big(\mathcal{D}^{re}([h^{re}, \tilde{h}]), y_j^{re}; \theta^{re}\big)$
11: update θ^{re} using ∇l^{re}
12: until convergence

h^{ner} and h^{re} are vector sequences. $\tilde{h}^{ner}$, $\tilde{h}^{re}$ and $\tilde{h}$ are vectors. Therefore, before concatenating $\tilde{h}$ with $\tilde{h}^{ner}$ or $\tilde{h}^{re}$, we expand $\tilde{h}$ to match the dimension.

For a NER instance (x_i^{ner}, y_i^{ner}), the BiLSTM layer $LSTM^{ner}$ takes x_i^{ner} as input and outputs h^{ner} (line 3). Then we use the attention method to generate a vector $\tilde{h}^{ner}$ (line 4). Similarly, h^{re} and $\tilde{h}^{re}$ can be generated from a RE instance (x_j^{re}, y_j^{re}) (lines 5-6). $\tilde{h}^{ner}$ and $\tilde{h}^{re}$ can be considered as the information summarizations for the current NER and RE training instances respectively. Then the information is linearly combined via $\tilde{h} = W_5 \cdot [\tilde{h}^{ner}, \tilde{h}^{re}]$, where W_5 is the parameter matrix to learn task relations. To utilize task-specific and shared information, the concatenation $[h^{ner}, \tilde{h}]$ or $[h^{re}, \tilde{h}]$ is fed into the decoder $\mathcal{D}^{ner}$ or $\mathcal{D}^{re}$. The loss l^{ner} or l^{re} is generated based on the objective function $\mathcal{L}$ (lines 8 and 10). Finally, the parameters θ^{ner} or θ^{re} are updated using the gradient ∇l^{ner} or ∇l^{re} (lines 9 and 11).

References

1. Chen X, Cardie C. Multinomial Adversarial Networks for Multi-Domain Text Classification. Proc 2018 Conf North Am Chapter Assoc Comput Linguist Association for Computational Linguistics; 2018. p. 1226–1240.

2. Duong L, Cohn T, Bird S, Cook P. Low Resource Dependency Parsing: Cross-lingual Parameter Sharing in a Neural Network Parser. Proc 53rd Annu Meet Assoc Comput Linguist Association for Computational Linguistics; 2015. p. 845–850.

3. Argyriou A, Evgeniou T, Pontil M. Multi-task Feature Learning. Proc 19th Int Conf Neural Inf Process Syst Cambridge, MA, USA: MIT Press; 2006. p. 41–48.

4. Misra I, Shrivastava A, Gupta A, Hebert M. Cross-Stitch Networks for Multi-task Learning. 2016 IEEE Conf Comput Vis Pattern Recognit CVPR 2016. p. 3994–4003. [doi: 10.1109/CVPR.2016.433]
