# Supplementary material for: Extraction of Information Related to Adverse Drug Events from Electronic Health Record Notes: Design of an End-to-End Model Based on Deep Learning
Source: JMIR Med Inform. 2018 Nov 26;6(4):e12159. doi: 10.2196/12159 (PMC6288593; doi:10.2196/12159)
Supplement: Multimedia Appendix 4 [file medinform_v6i4e12159_app4.pdf]

Multimedia Appendix 4: Experimental Settings

Hyper-Parameters

We split some data from the training set as the development set. Some hyper-parameters are tuned based on the development set and others are chosen empirically based on previous work [1,2]. The hyper-parameters in our models are shown in Table 1. For conciseness, the dimensions of some parameters such as W_1, W_2, W_3, W_4 and W_5 are not listed because they can be easily deduced from Table 1. Besides, the learning rate and batch size were set as 0.001 and 8. The L2 regularization parameter and dropout rate were set as 1e-8 and 0.5.

Table 1. Hyper-parameter settings.

Model	Hyper-parameter
NER	
	$w_n = 200;\ cap_n, pos_n, c_m = 20$
	$char_n = 50;\ h_n^{ner} = 200$
RE	
	$p_n^{e1}, p_n^{e2}, ew_1, ew_2, et_1, et_2, tn, en = 10$
	$h_n^{re}, h^{att} = 200$
HardMTL	$h^{share} = N \times 200$
RegMTL	$h_1^{ner}, h_2^{ner}, h_1^{re}, h_2^{re} = N \times 200$
LearnMTL	$\tilde{h}^{ner}, \tilde{h}^{re} = 200; \tilde{h} = 50$

Evaluation Metrics

Standard precision (P), recall (R), F1 were used to evaluate our models. An entity is counted as true-positive (TP) if its boundary and type are correct. A relation is counted as TP if its entity arguments are correct, meanwhile the relation type is correct.

Embedding

For the MADE dataset, we used pretrained word embeddings in the biomedical domain [3]. Other embeddings for the features such as POS tags, positions and entity types were randomly initialized with uniform distributions.

Preprocessing

The NLTK toolkit [4] was utilized for sentence splitting, tokenization and POS tagging. The words were not transformed into their lowercase forms since we need to keep the capital information. In the MADE dataset, there are approximately 20% relations whose entity arguments are not located in the same sentence. These inter-sentence relations lead to some problems for relation extraction. For example, we need to enumerate more entity pairs to determine whether they have relations. Moreover, many entities located in different sentences actually have no relations, so the enumeration may generate numerous

“None” relation instances. To avoid generating too much “None” relation instances, we limited the relation span to no more than 3 sentences. In addition, to further make the training data balanced, we down-sampled the “None” relation instances by randomly using 3% of them in each training epoch.

References

1. Yang J, Liang S, Zhang Y. Design Challenges and Misconceptions in Neural Sequence Labeling. Proc 27th Int Conf Comput Linguist Association for Computational Linguistics; 2018. p. 3879–3889.

2. Chen X, Cardie C. Multinomial Adversarial Networks for Multi-Domain Text Classification. Proc 2018 Conf North Am Chapter Assoc Comput Linguist Association for Computational Linguistics; 2018. p. 1226–1240.

3. Pyysalo S, Ginter F, Moen H, Salakoski T, Ananiadou S. Distributional semantics resources for biomedical text processing. 5th Int Symp Lang Biol Med 2013. p. 39–43.

4. Bird S, Klein E, Loper E. Natural Language Processing with Python: Analyzing Text with the Natural Language Toolkit. O’Reilly Media, Inc.; 2009. ISBN:978-0-596-55571-9
