# Supplementary material for: Extraction of Information Related to Adverse Drug Events from Electronic Health Record Notes: Design of an End-to-End Model Based on Deep Learning
Source: JMIR Med Inform. 2018 Nov 26;6(4):e12159. doi: 10.2196/12159 (PMC6288593; doi:10.2196/12159)
Supplement: Multimedia Appendix 5 [file medinform_v6i4e12159_app5.pdf]

Multimedia Appendix 5: Examples

Example 1
Relation: Medication-ADE, Entity1-Medication, Entity2-ADE
I do want to continue to hold the **[Velcade]$_{\textbf{Entity1}}$** as his **[peripheral neuropathy]$_{\textbf{Entity2}}$** continues to improve.

Example 2
Relation: Medication-Dosage, Entity1-Medication, Entity2-Dosage
MEDICATIONS: **[Ciprofloxacin]$_{\textbf{Entity1}}$** **[500 mg]$_{\textbf{Entity2}}$** p.o. daily, the rest of the medications are documented in the chart and were reviewed.

Example 3
Entity: Indication
ASSESSMENT : [** Name **] [** Name **] is a 55 - year - old male with : 1. lymphoplasmacytoid lymphoma with an **[IgG kappa monoclonal protein]$_{\textbf{Indication}}$.**

Example 4
Entity: SSLIF
PAST MEDICAL HISTORY : Non - Hodgkin lymphoma diagnosed in [** Date **] , monoclonal IgG kappa protein anemia , hypertension , prolonged hospitalization on [** Date **] for sepsis , **[necrotizing fasciitis due to Salmonella]$_{\textbf{SSLIF}}$.**

Example 5
Entity: ADE
Significant **[epistaxis]$_{\textbf{ADE}}$** requiring hospitalization in [** Date **] , which continues .

Example 6
Entity: Indication
Chronic **[back pain]$_{\textbf{Indication}}$** secondary to the lymphoma .

Example 7
Relation: Medication-ADE, Entity1-Medication, Entity2-ADE
His current therapy includes **[thalidomide]$_{\textbf{Entity1}}$** 50 mg a day for 2 weeks out of the month. He had been on Velcade, which was stopped secondary to increasing **[peripheral neuropathy]$_{\textbf{Entity2}}$**

Example 8
Relation: Medication-Indication, Entity1-Medication, Entity2-Indication
According to the patient, she is also taking **[Flovent]$_{\textbf{Entity1}}$**, ProAir and Spiriva. She is using Chantix to help her **[quit smoking]$_{\textbf{Entity2}}$**

Example 9
Relation: Medication-Indication, Entity1-Indication, Entity2-Medication

She had contacted the physician complaining of **[chest discomfort]**$_{\text{Entity1}}$ and gurgling in chest. She had increased edema and shortness of breath. She was advised to go to the emergency room, but refused. She took an additional dose of **[Lasix]**$_{\text{Entity2}}$ 40 mg and symptoms improved.
